# Retrospective analysis: checkpoint inhibitor accessibility for thoracic and head and neck cancers and factors influencing it in a tertiary centre in India

**DOI:** 10.3332/ecancer.2022.1464

**Published:** 2022-11-03

**Authors:** Vijay Patil, George Abraham, Madala Ravikrishna, Atanu Bhattacharjee, Vanita Noronha, Deevyashali Parekh, Nandini Menon, Jyoti Bajpai, Kumar Prabhash

**Affiliations:** Department of Medical Oncology, Tata Memorial Center and HBNI, Parel, Mumbai 400012, India; † These authors contributed equally

**Keywords:** accessibility, immunotherapy, checkpoint inhibitors, low & middle-income countries

## Abstract

**Background:**

Access to cancer care is an issue in low and low middle-income countries. The problem is worse with respect to access to new therapies like checkpoint inhibitors. Hence, we decided to audit our practice in the head and neck and thoracic medical oncology unit from 2015 to 2019 to study the accessibility of checkpoint inhibitors and factors influencing it.

**Methods:**

All patients who were registered in the head and neck and thoracic medical oncology unit between 2015 and 2019 were included in the study. Patients who received immunotherapy were identified from the prospective database of immunotherapy maintained by the department. We made a list of patients who were eligible for immunotherapy per year and identified how many of them received recommended immunotherapy. The indication for eligibility of immunotherapy was based on published pivotal data and it was applicable from the date of publication of the study online. Descriptive statistics were performed. For nominal and ordinal variable percentage with 95% confidence intervals (95% CI) was provided. Factors impacting the accessibility of immunotherapy were identified.

**Findings:**

A total of 15,674 patients were identified who required immunotherapy; out of them only 444 (2.83%, 95% CI: 2.58–3.1) received it. Among head and neck cancer patients, 4.5% (156 out of 3,435) received immunotherapy versus 2.35% (288 out of 12,239) among thoracic cancer patients (*p* < 0.001). Among the general category (low socioeconomic), 0.29% (28 out of 9,405 ) versus 6.6% (416 out of 6,269) among the private category (high socioeconomic) received immunotherapy (*p* < 0.001). While 3.7% (361 out of 9,737) among males versus 1.39% (83 out of 5,937) females received immunotherapy (*p* < 0.001). There was also a temporal trend seen in the accessibility of immunotherapy (*p* < 0.001).

**Conclusion:**

The accessibility of immunotherapy is below 3% in India. Patients with head and neck cancers, those registered as private category and male patients had higher access to this therapy. There was also a temporal trend observed suggesting increased accessibility over the years.

## Introduction

Checkpoint inhibitors have revolutionised the treatment of cancer. These drugs have improved life span and quality of life and on most occasions when used alone have had a lower rate of grade 3 or higher adverse events [1–10]. As a result in multiple situations, these have received approvals for use from regulatory authorities across the globe. Like with any new therapy, patients in high-income countries have more access to immunotherapy, whereas in low or low-middle-income countries (LMICs) access is limited to a small minority of patients.

Access to cancer care is an issue in low-income countries and LMICs [11]. This disparity in cancer care is further highlighted when we look at the access to new therapies in LMICs. Multiple new advances were seen in oncology in the last 2 to 3 decades such as robotic surgery, proton therapy and checkpoint inhibitor therapy. Among these checkpoint inhibitors are probably the only ones that have shown an overall survival benefit in multiple settings in randomised clinical trials [12–14]. Hence, having access to these life-saving medications is important. We work at a premier academic tertiary care centre in Mumbai in India. Our centre sees more than 35,000 new cancer patients per year which represents a significant cancer burden of the country. We decided to audit our data from 2015 to 2019 to study the access to checkpoint inhibitors in our setting and the factors that influence it.

## Methods

### Data collection

Patients receiving immunotherapy were identified from two databases: the immunotherapy database and the pharmacy database. The immunotherapy database is a prospective database of all patients receiving immunotherapy maintained by a subunit under ‘solid tumor’ in the Department of Medical Oncology at the Tata Memorial Center. The pharmacy maintains the pharmacy database at our centre and we identified all patients who had taken checkpoint inhibitors from this database. The data from these two databases were reconciled and from this reconciled database we extracted data of the patients who met the criteria below:

1. Adults aged > or = 18 years
2. Cancer site (one of the below)
  - Head and neck
  - Thorax
3. The time period between January 2015 and December 2019
4. Receiving Nivolumab, Pembrolizumab, Atezolizumab, Durvalumab, Avelumab or Ipilimumab.

Patients who had performance status 3 or greater and those that were unwilling for further treatment were excluded.

This data of immunotherapy recipients were collected as a part of a retrospective analysis approved by the Institutional Ethics Committee. Data regarding gender and patient category were extracted from these patients. Categories were defined as general or private. General and private categories in the institute are chosen by the patient. Patients who are willing to pay for their treatment, have personal insurance or have state-central government employee insurance fall in the private category. The rest were classified as a general category.

Patients eligible for immunotherapy – These patients were identified from individual outpatient department databases where available and from annual reports.

Medical Oncology maintains a meticulous annual report which categorises patients in detail as per the treatment received and by its indication. Thus, for each year, there was detailed data stratifying patients by the line of treatment received, best supportive care, Programmed cell death ligand (PDL) assumption, etc. We used the usage indication for each checkpoint inhibitor as per the respective landmark study (Table 1) and thus patients from our annual review database who met the usage criteria for these checkpoint inhibitors were included as ‘eligible’. These were patients who were offered systemic therapy. These were identified and subjected to the below-mentioned criteria listed.

1. Adult patients ( age > or = 18 years)
2. Cancer disease site (one of the below)
  - Head and neck
  - Thorax
3. The time period between January 2015 and December 2019
4. Patients for particular indications were considered eligible for immunotherapy from 1 month after the first publication of any positive checkpoint inhibitor data in abstract presentations or peer-reviewed journals. The table used for the identification of the patient pool is shown in Table 1.

In addition, the data for gender and category distribution were extracted from the hospital’s annual report.

### Statistical analysis

Descriptive statistics were performed. Percentage with its 95% confidence intervals (CI) is provided. Factors impacting access to immunotherapy were sought using the proportion test. A *p*-value of 0.05 was considered significant.

## Results

### Accessibility

A total of 15,674 patients were identified who required immunotherapy; of which only 444 (2.83%, 95% CI: 2.58–3.10) received it. The distribution of patients eligible as per cancer disease management group and time period is shown in Table 2. A temporal trend was seen suggesting an increase in the accessibility of immunotherapy over the years (*p* < 0.001, Table 2).

### Factors impacting accessibility

Factors affecting accessibility were the site of the disease, gender and socio-economic status of the patient. The percentage of patients who received immunotherapy was higher in head and neck cancers compared to thoracic cancer patients (4.5% (156 out of 3,435) versus 2.35% (288 out of 12,239) (*p* < 0.001)). More male patients received immunotherapy as compared to female patients (3.7% (361 out of 9,737) versus 1.39% (83 out of 5,937) (*p* < 0.001) (Table 3). Among the general category (low socioeconomic), 0.29% (28 out of 9,405) of patients received immunotherapy as compared to 6.6%(416 out of 6,269) of the private category (relatively high socioeconomic) patients (*p* < 0.001).

## Discussion

To the best of the authors’ knowledge, this is the first study evaluating the accessibility of checkpoint inhibitors from LMICs. The study shows a real-life picture of the access to these molecules in a LMIC. Less than 3% of patients at our centre can access checkpoint inhibitors. Hence, there is an urgent need to address the issue of accessibility. This accessibility is variable across broad disease management groups with the maximum still not crossing 5% and this was statistically significant. There was a significant difference in accessibility by gender and category of patient. As explained in the **Methods** section, private category patients have either higher socio-economic status or have access to insurance. Hence, the accessibility in this cohort was higher. However, in spite of accessibility being higher, it was below 10%. This suggests that even in affording patients or those with insurance, these drugs are largely inaccessible.

All our patients were from outside of clinical trials. We had a few clinical trials on checkpoint inhibitors ongoing at our centre however since the indications were not yet Food and Drug Administration (FDA) approved, the patients under those trials have not been included in this study. We do not have detailed demographic information for patients included in this specific study; however, we have the demographic details of patients that visit our centre for treatment. 50% of patients are from the state of Maharashtra while 50% are from states outside of Maharashtra. 20% of patients are from the city of Mumbai.

The cost of new medications, especially life-saving drugs, is a complex issue. The cost of these drugs is enormous [15–17] and unfortunately, there is a lack of guidelines suggesting fair pricing. The costs are kept similar across the globe and there is limited support for patients from LMICs. These costs which might be affordable for high-income countries are not necessarily affordable by LMIC. Hence, we are of the opinion that having differential pricing across the globe might help in improving accessibility. The presence of patient assistance programmes can further add to this context. The non-availability of these drugs is another issue influencing accessibility. The late introduction of drugs in LMICs is not uncommon. Drugs are either not introduced or introduced late in multiple low-income countries thus impacting their use. For example, Ipilimumab was launched in India after 2019 [18], while the seminal publication of this drug came in 2010 in melanoma [19].

Nivolumab received approval for lung cancer in India in June 2016 and for head and neck cancers in October 2017. Ipilimumab, as mentioned, only began being sold for use in October 2020. Pembrolizumab received approval for lung cancer in July 2018 and for head and neck cancers in September 2021.

In India, multiple government schemes support treatment [20]. Similarly, multiple non-governmental organisations or trusts also support patients’ needs. However, these schemes commonly do not support treatment that comes under the title of ‘palliative’ therapy irrespective of its benefit. For example, surgical treatments like Whipple’s surgery (for pancreatic cancer) [21] or pneumonectomy (locally advanced lung cancer) [22] where most of the patients fail within 1 year are supported as these treatments are labelled as ‘curative’. But, on the contrary, treatments like checkpoint inhibitors or even drugs like osimertinib in Epidermal Growth Factor Receptor (EGFR) mutated lung cancer [23] are not commonly supported. Even though the benefit of these drugs is much beyond the benefit of the above-mentioned procedures. Obviously, the high cost of these drugs prohibits government schemes and makes it challenging. Further, most government schemes are not flexible for the accommodation of patient assistance programmes. A combination of the decrease in drug cost, use of patient assistance programmes in government schemes and distribution of government resources on 2–5 year survival benchmarks might help in improving accessibility to these drugs. Other issues include drugs/pharmaceutical companies reaching LMICs relatively later as well as accessibility challenges with these drugs being available at pharmacies only in tier 1 cities in India, for example, and not in other cities.

Multiple insurances available in India do not support palliative treatment or treatment with targeted or immunotherapy [24] thus limiting the usefulness of such insurances in treating cancer patients. Additionally, this might further limit their accessibility [25]. There is an urgent need for national and international bodies to have a guideline on what treatments need to be reimbursed by insurance.

There is a need for positive steps from LMICs for addressing this disparity in the accessibility of high-cost anti-cancer drugs. The development of generic or bio-similar molecules and innovative studies on low-dose regimens or longer duration treatment schedules would help bring down the cost of treatment and improve accessibility in these regions. There are multiple retrospective analyses that suggest that such schedules might be helpful [26–28]. However, there is need for performing dose response, minimum effective dose finding studies and these need to be backed up by phase 2 and phase 3 randomised studies. Our team has completed one such randomised phase 3 study evaluating 20 mg Nivolumab in head and neck squamous cell carcinoma which suggested use of low-dose nivolumab in comparison with chemotherapy does improve outcomes. Our team is running another randomised study (CTRI/2020/02/023441) evaluating the role of altered schedules. If these studies meet their endpoints, then such schedules would help in improving accessibility. These and similar innovative strategies need to be undertaken in LMICs to combat this issue.

The study is not without its limitations. This was a single centre study and the data collection was retrospective. However, our centre is a premier centre in the country and nearly 50% of our patients are from regions outside where the centre is located. The data were limited to head and neck and thoracic malignancies as the investigator team largely works in this domain. Thus, our results on checkpoint inhibitor accessibility are limited to thoracic and head and neck cancers. A large multicentric international study might be able to cover a greater range of sites. Our study also wasn’t designed to evaluate if cost discrimination in different countries could potentially be a solution for low access.

The criteria used for immunotherapy were evolving over the course of the study period. As updates on indications of immunotherapy across lines of treatment came in over the study period, we updated the eligibility of patients for immunotherapy accordingly. The drugs used by site, intent and line of treatment are depicted in Table 4.

## Conclusion

The accessibility of immunotherapy is below 3% in India. Patients with head and neck cancers, those registered as private category and male patients had higher access to this therapy. There was also a temporal trend observed suggesting increased accessibility over the years.

## Funding

None.

## Conflicts of interest

The authors certify that they have no affiliations with or involvement in any organisation or entity with any financial interest (such as honoraria; educational grants; participation in speakers’ bureaus; membership, employment, consultancies, stock ownership or other equity interest; and expert testimony or patent-licensing arrangements) or non-financial interest (such as personal or professional relationships, affiliations, knowledge or beliefs) in the subject matter or materials discussed in this manuscript.

## Author contribution

Concept: All authors

Design: Dr Kumar Prabhash, Dr Vijay M. Patil

Literature search: All authors

Data acquisition: All authors

Data analysis: Dr Kumar Prabhash, Dr Vijay M. Patil

Statistical analysis: Dr Kumar Prabhash, Dr Vijay M. Patil

Manuscript preparation: All authors

Manuscript editing: All authors

Manuscript review: All authors

## Data sharing statement:

Data would be shared post-publication for a period of 5 years for scientific purposes on request, as per the rules and regulations of the Government of India. Data would be available on contacting Dr Kumar Prabhash at kumarprabhashtmh@gmail.com.

## Declaration of interest:

Vijay Maruti Patil

Research funding – AstraZeneca (Inst); Eisai Germany (Inst); Intas (Inst); Johnson & Johnson/Janssen (Inst); NATCO Pharma (Inst); Novartis (Inst).

George Abraham

No relationships to disclose.

Madala Ravikrishna

No relationships to disclose.

Atanu Bhattacharjee

No relationships to disclose.

Vanita Noronha

Research funding – Amgen (Inst); AstraZeneca (Inst); Dr. Reddy’s Laboratories (Inst); Intas (Inst); Sanofi/Aventis (Inst).

Travel, accommodations, expenses – American Society of Clinical Oncology.

Deevyashali Parekh

No relationships to disclose.

Nandini Sharrel Menon

No relationships to disclose.

Jyoti Bajpai

No relationships to disclose.

Kumar Prabhash

Research funding – Alkem Laboratories (Inst); BDR Pharmaceutics (Inst); Biocon (Inst); Dr Reddy’s Laboratories (Inst); Fresenius Kabi (Inst); NATCO Pharma (Inst); Roche (Inst).

## Figures and Tables

**Table 1.** Table depicting indication of checkpoint inhibitor applicable during the study period of January 2015 to December 2019.

Line of therapy	Landmark publication	Month and year from which patients considered eligible
Head and neck cancer – Squamous cell carcinoma		
Second-line	Ferris *et al* [1]. CheckMate 141	December 2016
First-line	Burtness *et al* [3]. Keynote 048	December 2019
Lung – Non-small cell lung cancer (NSCLC) (palliative)		
Second-line	Borghaei *et al* [4]. CheckMate 57	November 2015
First-line	Reck *et al* [5]. Keynote 024 TPS ≥ 50%. NSCLC – Non-EGFR/ALK	December 2016
First-line	Gandhi *et al* [6]. Keynote 189 Nonsquamous – Non-EGFR/ALK	June 2018
First-line	Paz-Ares *et al* [7], Keynote 407 Squamous NSCLC	December 2018
Lung – NSCLC (radical CTRT)		
CTRT	Antonia *et al* [8], PACIFIC Trial	December 2017
Lung – Extensive SCLC		
First-line	Horn *et al* [9]. IMpower133	January 2019
Oesophagus – Second line		
Second-line	Kato *et al* [10]. ATTRACTION-3	December 2019

ALK: Anaplastic Lymphoma Kinase; EGFR: Epidermal Growth Factor Receptor; TPS: Tumor Proportion Score

**Table 2.** Table depicting accessibility of checkpoint inhibitors from 2015 to 2019.

Disease site	Years	2015	2016	2017	2018	2019	*p*-value
Head and neck cancer	Number of patients with indication for immunotherapy	-	776	773	750	1136	<0.001
	Number of patients who received immunotherapy	-	2	27	43	84	
	Percentage of patients receiving immunotherapy	-	0.25	3.49	5.7	7.3	
	95% CI of the percentage of patients receiving immunotherapy	-	0.01–1	2.4-5.1	4.3–7.7	6–9.1	
Thoracic cancer	Number of patients with indication for immunotherapy	1,365	1,844	2,730	3,000	3,300	<0.001
	Number of patients who received immunotherapy	1	3	44	110	130	
	Percentage of patients receiving immunotherapy	0.07	0.16	1.6	3.6	3.9	
	95% CI of the percentage of patients receiving immunotherapy	0–0.4	0.03–0.5	1.2–2.1	3.1–4.4	3.3–4.7	

**Table 3.** Table depicting factors impacting the accessibility of checkpoint inhibitors.

Variable	Number of patients who received immunotherapy	Number of patients who received immunotherapy (%)	The number of patients who did not receive immunotherapy (n)	The number of patients who did not receive immunotherapy (%)	Total number of patients	*p*-value
Site of disease						
Head and neck carcinoma	156	4.5	3,279	95.5	3,435	<0.001
Thoracic cancer	288	2.4	11,951	97.6	12,239	
Gender						
Male	361	3.7	9,376	96.3	9,737	<0.001
Female	83	1.4	5,854	98.6	5,937	
Socioeconomic status						
General	28	0.3	9,377	99.7	9,405	<0.001
Private	416	6.6	5,853	93.4	6,269	
Age						
Elderly^a^	77	2.9	2,634	97.1	2,711	1.000
Non-Elderly	367	2.9	12,596	97.1	12,963	
Stage						
III	15	14.9	101	83.1	116	<0.001
IV	429	2.8	15,129	97.2	15,558	

a Elderly was defined as age > 65 years

**Table 4.** Table depicting the site, intent, lines of treatment and drugs used.

Site	Intent	Line of treatment	Drugs
Head and neck	Palliative	1st line, 2nd line and beyond	Nivolumab Pembrolizumab
NSCLC	Palliative	1st line, 2nd line and beyond	Nivolumab Nivolumab + Ipilimumab Pembrolizumab Atezolizumab
NSCLC	Curative	-	Durvalumab
SCLC	Palliative	1st line	Atezolizumab
Oesophagus	Palliative	2nd line and beyond	Nivolumab

NSCLC, Non-small cell lung cancer; SCLC, Small cell lung cancer
